# Human Adipose Mesenchymal Stem Cell-derived Exosomes Protect Mice from DSS-Induced Inflammatory Bowel Disease by Promoting Intestinal-stem-cell and Epithelial Regeneration

**DOI:** 10.14336/AD.2021.0601

**Published:** 2021-09-01

**Authors:** Hongliang Yu, Xudong Yang, Xian Xiao, Meiqian Xu, Yanlei Yang, Chunling Xue, Xuechun Li, Shihua Wang, Robert Chunhua Zhao

**Affiliations:** ^1^Institute of Basic Medical Sciences Chinese Academy of Medical Sciences, School of Basic Medicine Peking Union Medical College, Beijing, China.; ^2^Department of Cell Biology, School of Life Sciences, Shanghai University, Shanghai, China

**Keywords:** Inflammatory bowel disease (IBD), Human adipose mesenchymal stem cell (hADSC), Exosome, intestinal stem cell (ISC), epithelial cell

## Abstract

Inflammatory bowel disease (IBD) remains a severe disease for most patients, with its incidence and prevalence increasingly globally. Currently, there is no effective treatments for IBD, and traditional treatments have multiple side effects. Therefore, novel therapeutic strategies or alternative drugs are urgently needed. Previous studies have shown that mesenchymal stem cell-derived exosomes have exhibited promising therapeutic effects on inflammatory disease. Here, we performed intravenous injection of human adipose mesenchymal stem cell (hADSC)-derived exosomes (hADSC-Exo) in a DSS-induced IBD mouse model and found that hADSC-Exo promoted functional recovery, downregulated inflammatory responses, reduced intestine cell apoptosis, increased epithelial regeneration and maintained intestinal barrier integrity. Moreover, we established a colon organoid, hADSC-Exo and TNF-α co-cultured system to explore the protective effect of hADSC-Exo on integrity of intestine mucosa and epithelial regeneration. We showed that hADSC-Exo not only can promote the proliferation and regeneration of Lgr5^+^ ISCs and epithelial cells but also ameliorate the inflammation damage in TNF-α induced inflammatory damaged mice colon organoids. Taken together, our findings indicate that hADSC-Exo protects intestine integrity, activates intestine epithelial cell and ISCs proliferation, suggesting that hADSC-Exo might be a potential effective treatment approach for IBD. We also provide a theoretical basis for new therapeutic strategies for cell-free therapy in inflammatory bowel disease.

Inflammatory bowel disease (IBD) including Crohn’s disease (CD) and ulcerative colitis (UC) is a chronic, nonspecific inflammatory disorder, which severely threatens the human health [1, 2]. IBD causes destruction of epithelial lining of the gut, which greatly impairs the life quality of most patients. Multiple causes, such as environmental, genetic, and inflammatory factors, may contribute to pathogenesis of IBD. Among these factors, epithelial dysfunction and crypt destruction play a defining role in the process of IBD [3, 4]. Despite recent progress in IBD research, there is still no effective clinical treatment strategy for IBD. Conventional treatment options for IBD include anti-inflammatory medications (5-amino salicylic acid, steroids) and immune-suppressants, which have limited therapeutic efficacy. However, their non-specific actions on immune system could result in allergic reactions, nausea, pancreatitis, and other side effects, highlighting the unmet need for novel treatment options for IBD [5, 6].

Mesenchymal stem cells (MSCs) have great potential in injury repair [7], autoimmune diseases [8], cardia-cerebrovascular diseases, and nervous system diseases [9, 10]. These cells possess self-renewing ability and capacity to differentiate into three lineages [11]. MSCs-based therapies have also exhibited great potential in treatment of IBD both in animals [12] and human individuals [13, 14]. Although MSC therapy has unique advantages and significant treatment efficiency as a novel therapeutic option for IBD, it still has its own limitations in terms of safety. Previous preclinical trial has indicated that only a few percentage of implanted mesenchymal stem cells engraft into injured tissues after their transplantation[6, 15], suggesting that therapeutic effects may be induced by bioactive molecules from MSCs. Compared to MSCs, MSC-derived exosomes (MSC-Exo) have similar functions of MSC without the defects of safety limitations.

Recent studies revealed that MSC-Exo had injury repair functions in several diseases [16, 17]. For example, Chen et al. reported that exosomes from human adipose mesenchymal stem cells promoted recovery from traumatic brain injury by suppressing microglia/ macrophage activation. Zhao et al. demonstrated that adipose MSC-Exo alleviated adipose inflammation and obesity through polarizing M2 macrophages [18]. Sun et al. found that human MSC-Exo attenuated type 2 diabetes mellitus via reversing peripheral insulin resistance and relieving β-Cell destruction [19]. Liu et al. demonstrated that bone-marrow MSC-Exo reduced murine colonic inflammation by downregulating inflammatory responses in a macrophage-dependent mechanism [6]. MSC-Exo can carry the proteins, RNAs, or lipids of MSCs and transfer them into target cells to fulfill their functions [20]. MSC-Exo have ideal properties such as non-oncogenicity, ultrastability, tissue specific homing [6, 21].Thus, MSC-Exo therapy as a cell-free option could be an alternative method for mesenchymal stem cell-based therapies in the long run.

In the present study, exosomes were isolated from human adipose mesenchymal stem cells. Using hADSC-Exo as a proof- of - concept testing, we performed tail intravenous injection of hADSC-Exo in a DSS-Induced IBD mouse model. We demonstrated that hADSC-Exo alleviated disease progression in IBD mice by promoting the proliferation and regeneration of Lgr5+ ISCs and epithelial cells. Exosomes can also be taken up by intestinal epithelial cells (HcoEpic cell line) and promote their proliferation in vitro. Moreover, we established a colonic organoid injury model induced by TNF-α and found that hADSC-Exo can not only promote the proliferation and renewal of colonic organoid, but also endow colonic organoid with the ability to resist inflammatory injury. In summary, our findings may lead to the development of novel and safe therapies for IBD, as well as opening up a new avenue for cell-free therapy.

## MATERIALS AND METHODS

### Cell culture

Cell line Hcoepic was obtained from American Type Culture Collection. Hcoepic was cultured in DMEM containing 10% fetal bovine serum (FBS). Human adipose tissues were obtained from the Department of Orthopaedics of Peking Union Medical College Hospital (Beijing, China), approved by the Chinese Academy of Medical Sciences and Peking Union Medical College. MSCs were isolated and culture-expanded from healthy volunteers as previously reported [22]. Stem cells were isolated from human adipose tissue and cultured in Dulbecco's modified Eagle's medium (DMEM)/F-12 supplemented with 2% fetal bovine serum, necessary growth factors, 100 U/mL penicillin and 100 μg/mL streptomycin. Cells were maintained at 37 °C in a humidified incubator with 5% CO2 and passaged with trypsin/EDTA after reaching confluence [23].

### Animal studies

All animal experiments were approved by the Institutional Animal Care and Use Committee of the Academic Committee of the Chinese Academy of Medical Sciences and Peking Union Medical College Hospital. The animal studies were performed as described previously [2, 5, 24]. Female C57BL/6 (6-8 weeks) mice were housed under specific pathogen-free conditions. To establish colitis mouse model, 3%(w/v) dextran sodium (DSS) was administrated in drinking water for mice, mice received either regular drinking water (control) or DSS drinking water (model) for 8 days. Mice in the hADSC-Exo treatment group were also tail vein administered hADSC-Exo (300 ug/mice) every 3 days during this study period. Physical activity, body weight, and feces were monitored every day. 8 days later, mice were euthanized and organs, plasma of interest were harvest for subsequent measurements.

### Isolation and characterization of hADSC-Exo

For extraction of hADSC-Exo, according to our previously described methods [25]. Briefly [26], hADSC were cultured with serum-free DF12 medium for 24 h. Then, the culture medium was collected and centrifuged at 3,000 rpm for 20 min, next, the medium was subjected to filtration on a 0.1-mm-pore membrane filter (Merck millipore) to remove cell debris and large vesicles. Finally, the supernatant was ultracentrifuged at 120,000 × g for 1.5 h and the pellet was suspended in PBS.

### Transmission electron microscopy

For TEM analysis of exosomes, Purified exosomes were fixed in 1% glutaraldehyde in PBS (pH 7.4). 2 µL drop of exosome suspension was transferred onto a Formvar-carbon coated electron microcopy grid, negatively stained with 3% (w/v) aqueous phosphotungstic acid for 1 min, and then examined using TEM [23, 27].

### Nanoparticle tracking analysis

hADSC-exo after ultracentrifugation were resuspended in PBS. Exosome sizes distribution and concentration were measured by ZetaView instrument (Particle Metrix) following the manufacturer’s instructions. Generally, the exosomes were diluted into 100-400 times with PBS [28].

### Western blotting

Cell exosomes were lysed in a western blotting lysis buffer-radioimmunoprecipitation (RIPA) with PMSF, then, the protein concentrations were quantified with a bicinchoninic acid (BCA) protein assay kit (Beyotime Biotechnology, Shanghai, China) [29]. Proteins were separated using 10% sodium dodecyl sulfate-polyacrylamide gel electrophoresis (SDS-PAGE), and subsequently electro-transferred onto a PVDF membranes, and the membranes were incubated in following antibodies: CD9 (rabbit IgG, proteintech), TSG101 (rabbit IgG, Abcam), HSP70 (rabbit IgG, SBI), Calnexin (rabbit IgG, CST), Alix (rabbit IgG, proteintech), GAPDH (rabbit IgG, proteintech), GAPDH was used as internal control. followed by chemiluminescent detection (Tanon, China) [30].

### Exosome labeling and uptake assay in vitro

hADSC-exo were stained with the dye,DiI (Invitrogen, C7000) according to the manufacturer's protocol. Generally, 1 µl Dye solution (1 mM) was added to hADSC-exo solution dissolved in 1 ml PBS, and the mixture was incubated for 20 min at 37 ?, then removed the free dye by ultrafiltration. Exosome - DiI mixture co-cultured with Hcoepic for 4, 12, 24, and 48 h. Excessive exosomes were then washed with culture medium, and the cell nuclei were stained with Hoechst 33342 (1:1000; Sigma-Aldrich) and then screened Hcoepic cells by fluorescence microscopy [26].

### Cell proliferation assay

Hcoepic epithelial cell proliferation assay was performed by cell counting kit-8 (CCK8, Dojindo Molecular Technologies) following manufacture instructions. Briefly, cells were seeded into 96 - well plates at a density of 1 ×10^4^ cells per well and incubated 24 h. Cells were then incubated with different concentration of hADSC-Exo (0, 0.1, 2, 5, 8, 10 µg/ml) in PBS for 24 and 48 h, after which the hADSC-Exo containing medium was removed and cells were thoroughly rinsed once with cold PBS, and then 10 µl of CCK8 was added to each well. The microplates were incubated at 37 ?, 5% CO_2_ for 2-4 h. thereafter, absorbance was read at 450 nm by use of a microplate reader. Untreated wells were used as a negative control [31].

### Serum cytokine analysis

Multiplex kits for measuring cytokines were purchased from Bio-Rad (Bio-Plex Pro Mouse Cytokine Grp I Panel 23-plex). The cytokine analyses were performed in accordance with the manufacturer's instructions for the xMAP technology with multiplex beads. Plates were measured using the Bio-Plex MagPix System and analysed with the Bio-Plex Manager version 6.1 (Luminex, Austin, TX, USA), agented by Wayen Biotechnologies (Shanghai, China), The following cytokines IL-6, IL-12, IL-1β, IL-13 and TNF-α were measured. Results were expressed as picograms per milliliter [32].

### Histological analyses of tissue sections by hematoxylin and eosin staining (H&E)

Mice colons collected from the animal studies were fixed in 10% neutral formalin at room temperature 1h or longer, then embedded in paraffin and then cut the samples into 6 um thickness sections and stained with hematoxylin and eosin (H&E). Stained slides were viewed and acquired using the pathological section scanner [33].

### Immunofluorescence staining

At eighth day after DSS water implementation, mice were euthanized, collected their colon and the tissues were fixed in 10% neutral formalin at room temperature 1h or longer, then embedded in paraffin and the samples were cut to 4 μm thickness sections, These 4-μm sections were deparaffinized, after that, the sections were blocked in 0.1 M Tris-HCl, 0.15 M NaCl, 2% FBS for 30 min and the primary antibody was used overnight at 4 ?. The sections were then washed with TNT buffer, incubated with secondary antibodies for 1 h at room temperature and mounted with Pro-Long Diamond antifade mountant containing DAPI [28].

### Scratch wound healing assay

Hcoepic cells were seeded in six-well culture plates at the density of 1 ×10^5^ cells per well at 37 ?, 5% CO^2^ overnight to allow the formation of confluent monolayer to 90% confluence [34]. Thereafter, cell monolayers were scratched with 200 µl pipette tip carefully to make wounds. Detached cells were rinsed of three times with PBS. Then, the cell monolayers were incubated in serum-free DMEM with 200 µg/ml hADSC-Exo for 48 h. Cells were photographed at 0, 4, 6, 12, 24, 48 h after wounding. Distances of wound area were quantified. All experiments were carried out in triplicates [35].

### Apoptosis and cell cycle assay by flow cytometry

Apoptosis of Hcoepic cell line was detected by flow cytometry with an Annexin V- Alexa Fluor 488/PI kit (Yeasen, China), according to the manufacture’s instruction. Briefly, after cells suspended in binding buffer, 5 μl Annexin V-Alexa Fluor 488 and 10 μl PI solution were added for 10 min in the dark at room temperature. Thereafter, the apoptosis was detected by use of a flow cytometer. Cell cycle assay for Hcoepic cells was detected with Cell Cycle Analysis Kit (Yeasen, China), according to the manufacture’s instruction. Generally, Cell precipitation was gently mixed with 1 mL pre-cooled 70% ethanol and fixed at 4? for more than 2 h or overnight. Next, the cells were precipitated by centrifugation at 1000 g for 5 min and resuspended with 1 mL pre-cooled PBS. Then, the cells were centrifuged again at 1,000 g for 5 min to precipitate. Add 0.5ml binding buffer containing 10 µl PI solution and 10 µl RNase A to each sample and incubated at 37 ? for 30 min without light. Thereafter, these samples were detected by use of a flow cytometer.

### Mouse colon crypt isolation and organoid culture

Colon organoids were derived from the colon of 4 to 6-week-old C57BL/6 mice, as described previously [4, 36]. For crypt isolation and organoid culture, we used organoid intestiCult^TM^ growth medium (Stemcell, 06005), according to the manufacture’s instruction. Briefly, the mouse colon was isolated and flushed cleanly with cold and opened longitudinally with scissor. Splay open the colon segment, use a pipettor to wash gently, use forceps to move the segment and apply clean buffer to rinse thoroughly. Using scissors cut the colon into 2 mm segments and put them into a new 50 ml tube containing 15 ml cold PBS, use a pre-wetted pipette to wash the colon pieces by pipetting up and down 3 times, let colon pieces settle by gravity and carefully remove the supernatant. Add 15 ml PBS and repeat this wash procedure 15-20 times until the supernatant is clear. Remove supernatant and incubated the colon segment in Gentle Cell Dissociation Reagent (Stemcell, 07174), at room temperature 20 min, resuspended the colon pieces in 10 ml of cold PBD with 0.1% BSA. The colon fragment fractionated, and crypt-containing fractions were passed through a 70um cell strainer for plating in Matrigel. Crypt- Matrigel suspension was allowed to polymerize at 37 ? for 15 min, colon organoids were grown in intestiCult^TM^ growth medium.

### Establishing co-cultured system containing TNF-α, hADSC-Exo and colon organoid

Colon organoids were seeded in 24-well plate and cultured with 750 µl intestiCult^TM^ growth medium at 37 ?, 5% CO^2^. The culture medium was changed every 3 days. After 6 days, hADSC-Exo (100 µg/ml) was added into culture medium, colon organoids were photographed at 48 h and 72 h after hADSC-Exo treated. Exosomes exposed for 72h later, TNF-α (40 ng/ml) was added into the culture medium, colon organoids were photographed at 40 h after TNF-α exposed using a Fluorescence microscope. All data analyses were presented using GraphPad Prism Software 7.0.

### Statistical analysis

All data are presented as the mean ± standard deviation (SD), comparisons between groups were analyzed via Student’s t test. Differences were considered statistically significant when *P < 0.05, **P < 0.01, and ***P < 0.001. Experments *in vitro* such as CCK8, western blotting, wound healing assay, etc. were routinely repeated at least three times. The animal weights were used for randomization and group allocation.

## RESULTS

### Characterization of hADSC and hADSC-Exo

**Figure 1.**
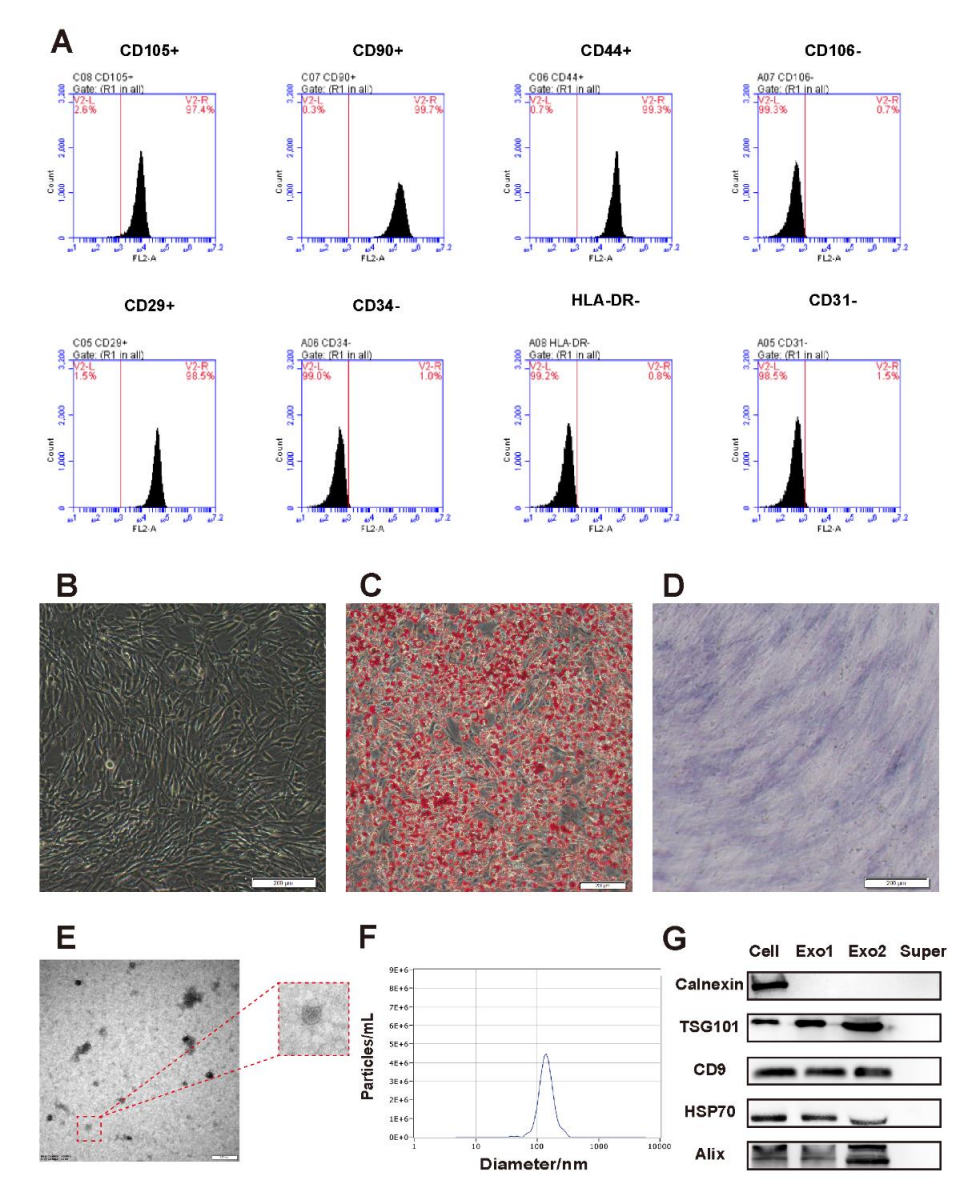
**Identification of hADSC and hADSC-Exo**. (**A**) Identification of hADSC surface markers through flow cytometry (CD-105, CD-90, CD-144, CD-106, CD-29, CD-34, HLA-DR, CD-31). (**B**) Microscopy of hADSC (Light sight, scale bar=200 µm). (**C**) Differentiation capacity of hADSC was demonstrated by Oil Red O staining for adipocytes. (Scale bars=200 um). (**D**) Differentiation capacity of hADSC was demonstrated by ALP staining for osteoblasts (Scale bars=200 µm). ALP, alkaline phosphatase. (**E**) Transmission electron microscope detection of hADSC-Exo (scale bar=200 nm). (**F**) NanoSight Nanoparticle Tracking analyzer detection of hADSC-Exo. (**G**) hADSC-Exo related surface markers were detected by western blotting analysis in hADSC and hADSC-Exo (Exo1, Exo2 derived from two different donors).

Flow cytometric analysis revealed that hADSC was positive (≥ 95%) for CD105, CD90, CD44, CD29, but negative (≤ 3%) for CD106, human leukocyte antigen DR (HLA-DR), CD34, CD31 (Fig. 1A). hADSC represented a classical fibroblast-like morphology and could differentiate into adipocytes or osteoblasts under specific culture conditions (Fig. 1B, C, D). We isolated hADSC-Exo from the supernatants of hADSC using ultra-centrifugation, and exosome morphology and size distribution were examined by transmission electron microscopy (TEM) and Nanoparticle tracking analysis (NTA). The extracted hADSC-Exo presented a typical cup-shaped morphology (Fig. 1E). The majority of exosomes had an expected diameter of around 100 nm, and peak diameter of hADSC-Exo is 132 nm (Fig. 1F). Furthermore, western blot results showed that hADSC-Exo expressed exosome markers such as tumor susceptibility gene 101 (TGS101), CD9, heat-shock protein 70 (HSP70) and Alix (Fig. 1G).

**Figure 2.**
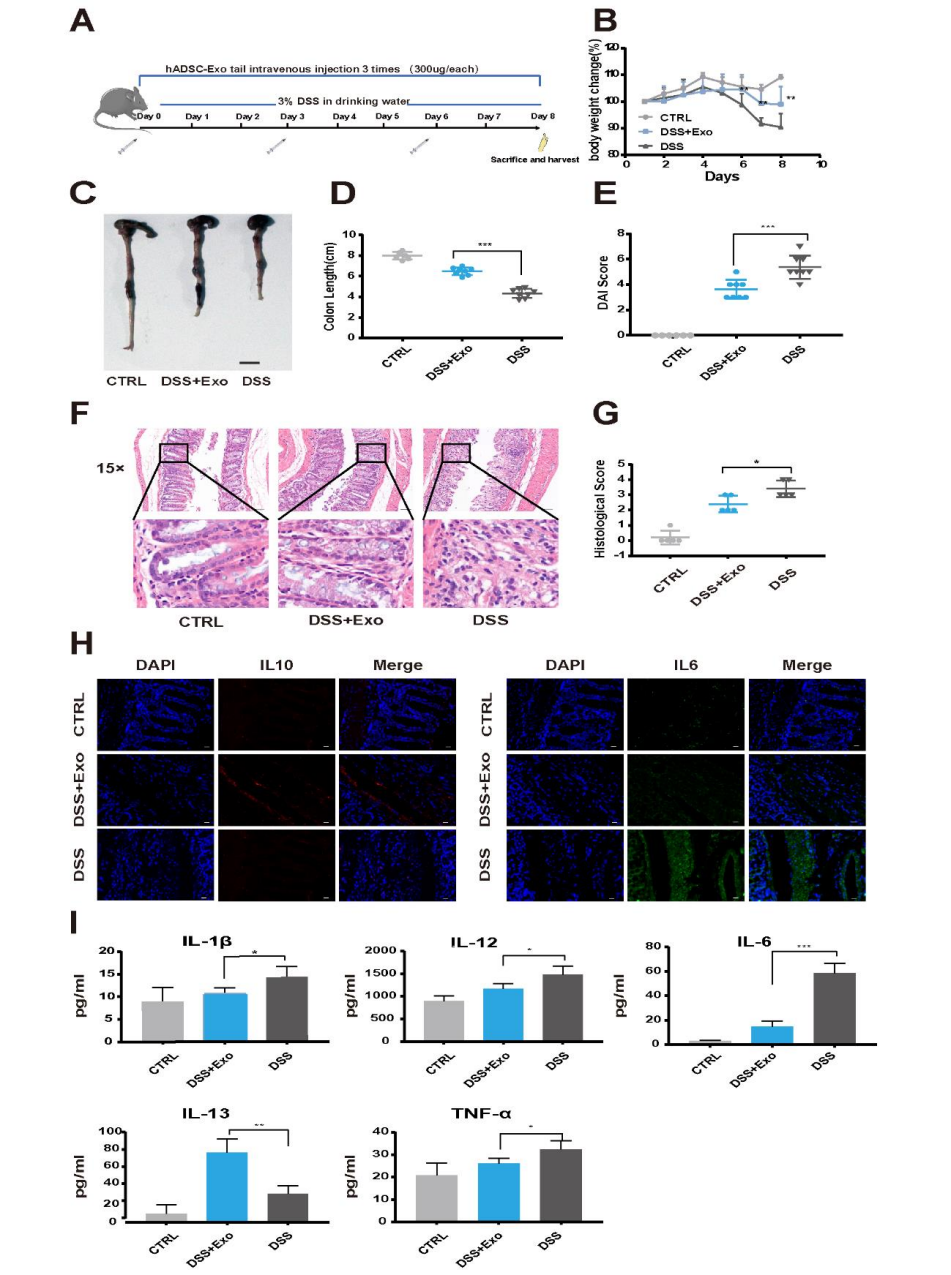
**hADSC-Exo relieves DSS-induced mouse IBD**. (**A**) Schematic diagram for DSS-induced IBD in C57BL/6 mice and hADSC-Exo administration. B) Changes in mice body weight. (C and D) Colon appearance and length in mice. (**E**) Disease activity index scores of mice. (F and G) H&E staining and histological scores of mice colon tissue (scale bar=100 µm, 15 ×; scale bar=50 µm, 40×). *P<0.05, **P<0.01. ***P<0.001. (**H**) Expression of IL-10 and IL-6 in colon tissue from mice was analyzed by immunofluorescence staining (scale bar=20 µm). (**I**) Expression of IL-1β, IL-12, IL-6, IL-13 and TNF-α in mice serum was detected by use of Multiplex ELISA kits (n=7, per group).

**Figure 3.**
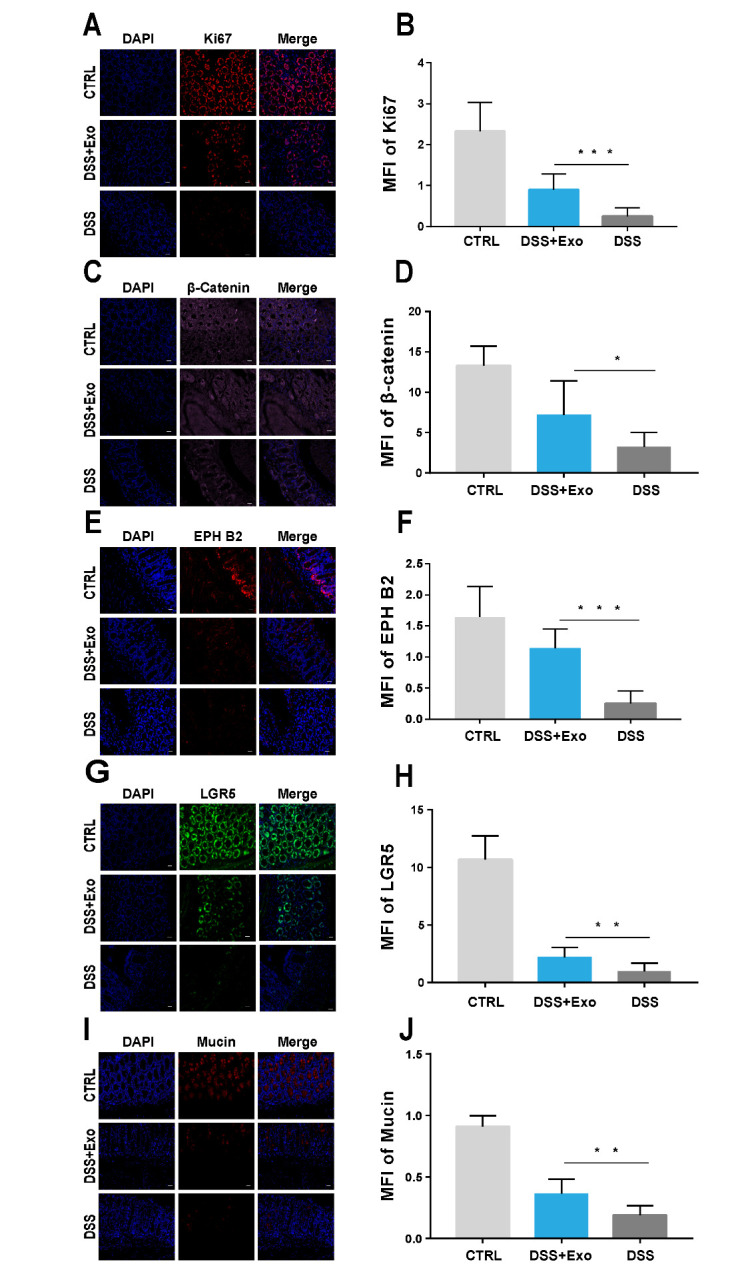
**hADSC-Exo reshape the cell structure in colon crypts of DSS-induced mice IBD**. (**A-D**) Immunofluorescence staining analysis of expression of epithelial cells proliferation related specific marker Ki67 and β-Catenin in mice colon tissue sections (scale bar=20 µm; n=7, per group). (**E-H**) Immuno-fluorescence staining analysis of expression of colon stem cells specific markers EPH B2 and LGR5 in mice colon tissue sections (scale bar=20 µm; n=7, per group). (I and J) Immuno-fluorescence staining analysis of expression of goblet cells specific marker Mucin in mice colon tissue sections (scale bar=20 µm; n=7, per group).

### hADSC-Exo inhibits colon injury and ameliorates DSS-induced mouse IBD

In order to determine the biological distribution of hADSC-Exo in mice after injection and whether hADSC-Exo can home to the inflammatory site of colon, we labeled hADSC-Exo with DIR dye and injected it into mice via tail vein. 12 h after injection, we detected the fluorescence by IVIS spectrum. The results showed that a large area of fluorescence could be seen in the abdominal cavity after 12 h injection of hADSC-Exo. Afterwards, we sacrificed the mice and analyzed the fluorescence in the intestine, and clear fluorescence can be seen in the intestine, including the colorectal tissue. The results showed that hADSC-Exo can home to the inflammatory site of colon (Supplementary Fig. 1).

**Figure 4.**
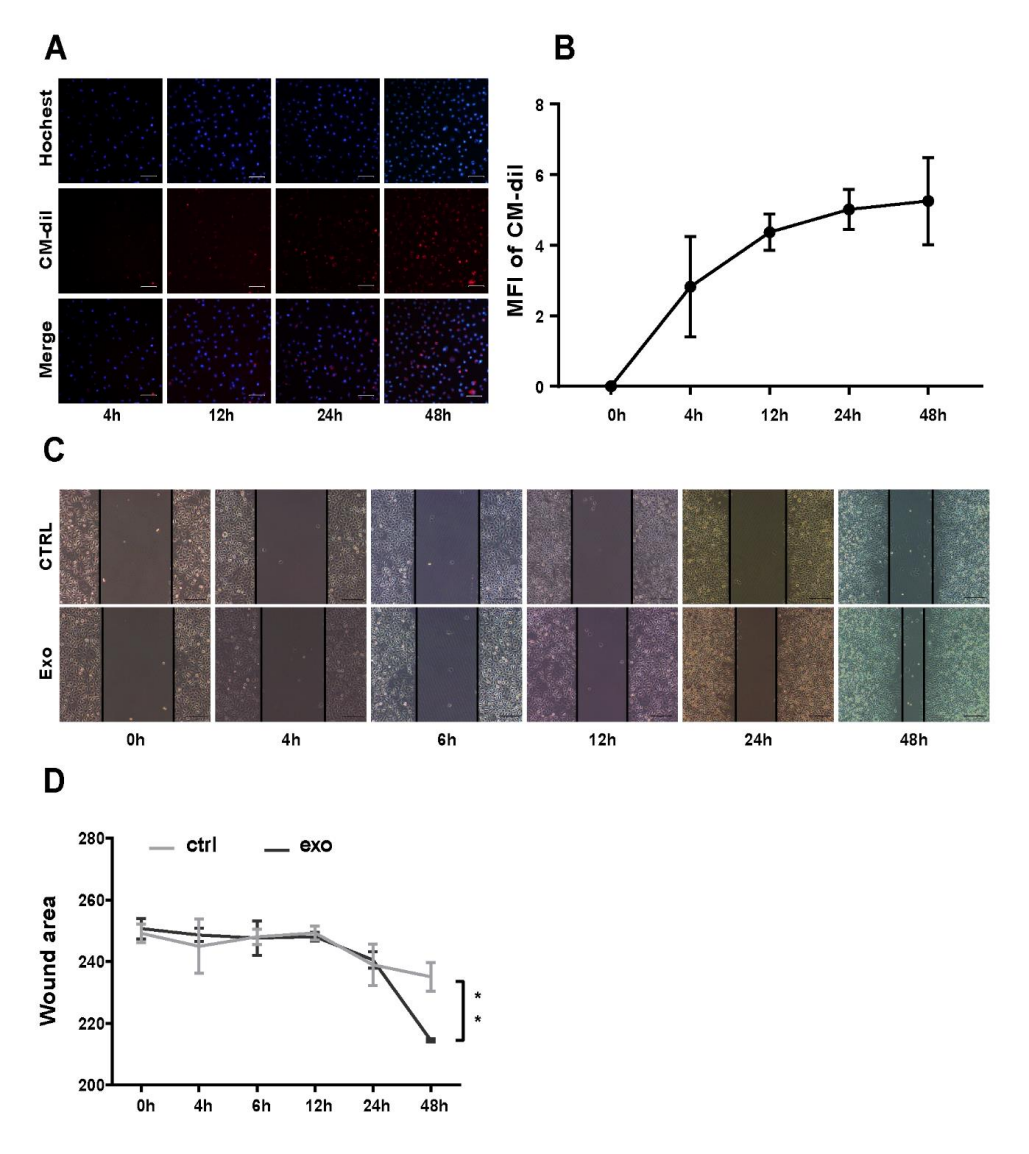
**hADSC-Exo up-taken by Hcoepic cells and promotes wound healing of Hcoepic cells**. (**A**) Uptake of hADSC-Exo by Hcoepic cells at 4, 12, 24, 48 h after co-culture. (**B**) Mean fluorescence intensity (MFI) of Dil in Hcoepic cells. (C and D) hADSC-Exo promotes the migratory activity of Hcoepic cells and accelerate wound healing, at 0, 4, 6, 12, 24, 48 h after co-culture.

**Figure 5.**
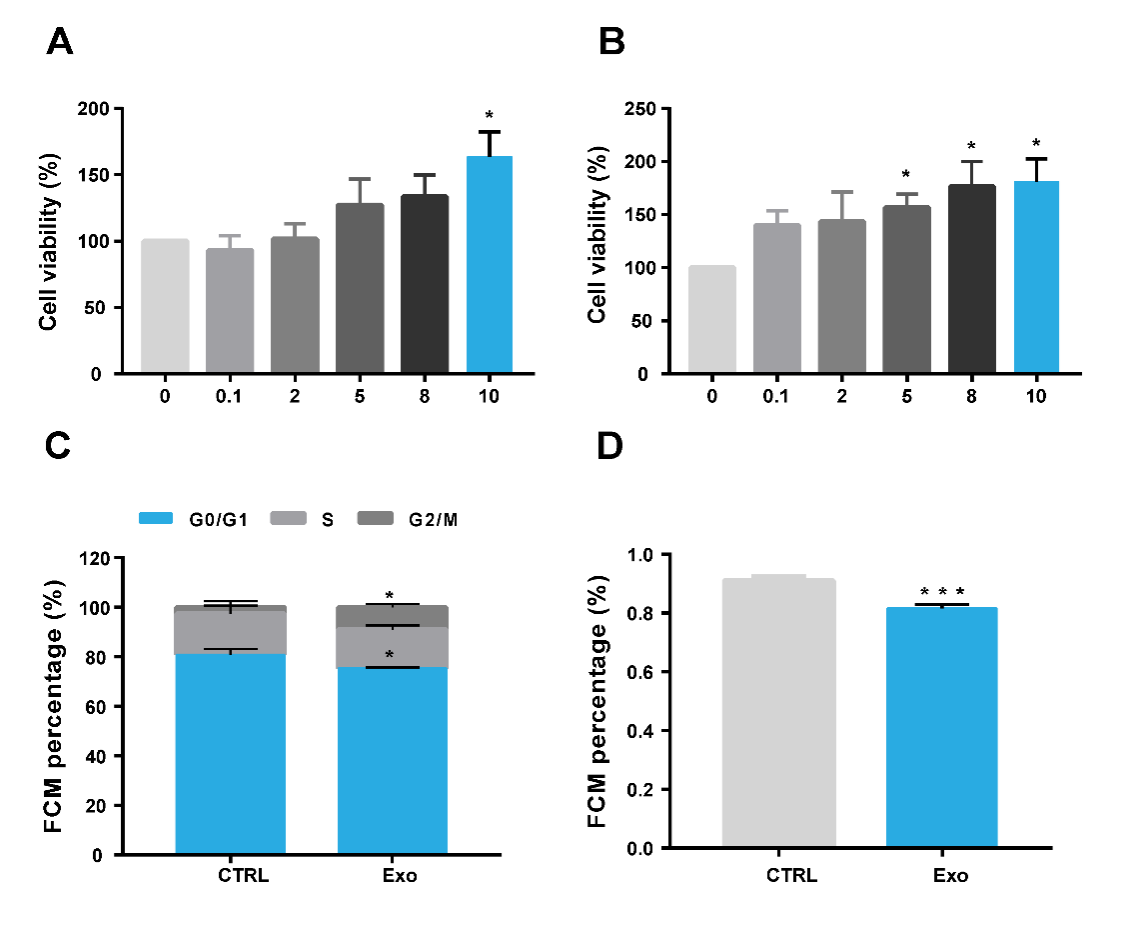
**hADSC-Exo facilitates Hcoepic cells proliferation, promotes cell cycle and inhibits cell apoptosis**. (A and B) CCK8 assay analysis the exosomes’ influences on the proliferation of Hcoepic cells at 24 (A) & 48 h (B). exosome concentration: 0, 0.1, 2, 5, 8, 10 µg/ml. (**C**) Exosomes’ influences on cell cycle of Hcoepic cells after hADSC-Exo administration 48 h at exosome concentration: 200 ug/ml. (**D**) Exosomes’ influences on cell apoptosis of Hcoepic cells after hADSC-Exo administration 48 h at exosome concentration: 200 µg/ml (All experiments repeated at least 3 times).

To determine the potential therapeutic effects of hADSC-Exo in injury tissues of IBD mouse. we established a DSS induced mouse model (a well-established mice model for study of human IBD), and we injected hADSC-Exo into these mice through the tail vain (Fig. 2A). The survival status, body weight and feces of mice were observed every day. By 8^th^day, the mice were sacrificed, and colon and serum were harvested for further analysis. Colon length changes, body weight loss and rectal bleeding are the typical features of IBD. As shown in Figure 2B, mice in DSS-induced IBD model group exhibited dramatic body loss, but hADSC-Exo significantly attenuated the loss of body weight. In DSS induced model group, 3% DSS drinking water led to colon shortenings. Nevertheless, hADSC-Exo administration significantly relieved colonic inflammation, increased the colon length (Fig. 2C, D). Tail vain injection of hADSC-Exo dramatically reduced disease activity indexes (DAI) scores during the disease progression (Fig. 2E). The histological effects of hADSC-Exo on DSS-induced colon inflammation and the severity of colonic ulceration were further examined by H&E. The results revealed that hADSC-Exo administrated mice maintained relative colonic structural integrity with no apparent ulceration. They also had less crypt loss and less inflammatory cell infiltration and lower histological scores (Fig. 2F, G).

The production of pro-inflammatory factors and anti- inflammatory mediators, such as IL-1β, IL-6, IL-12, TNF-α, IL10 and IL-13, play a critical role in the progression of IBD. To determine the effects of hADSC-Exo on the cytokines in colon tissues and mouse peripheral blood serum, we performed immune-fluorescence staining experiments in colon tissues and cytokine analysis with Multiplex kits in mouse peripheral blood serum. The immunofluorescence staining of colon tissue indicated that hADSC-Exo significantly reduced the pro-inflammatory factors IL-6 and promoted the production of anti- inflammatory mediators IL- 10 (Fig. 2H). Cytokine analysis in mouse peripheral blood serum results presented that hADSC-Exo significantly reduced the pro-inflammatory factors (IL-1β, IL-6, IL-12, TNF-α), while promoted the production of anti- inflammatory mediators IL-13 (Fig. 2I).

**Figure 6.**
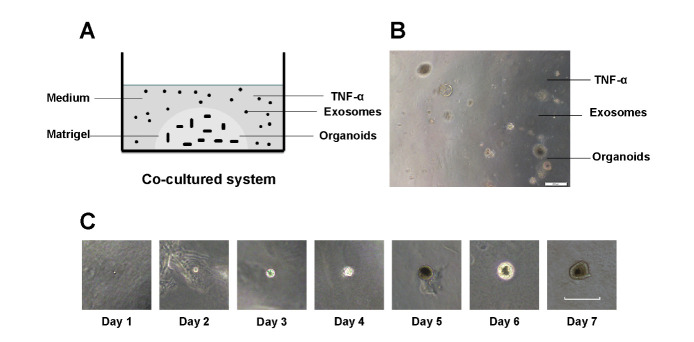
**Establishing co-cultured system containing TNF-α, hADSC-Exo, and colon organoids**. (**A**) The co-cultured model of TNF-α, hADSC-Exo, and colon organoids. (**B**) Organoids co-cultured with TNF-α and hADSC-Exo were photographed in light sight with microscope (scale Bar=200 um). (**C**) Growth status of the colon organoids in 7 days was observed under microscope (scale Bar=200 µm).

### hADSC-Exo attenuate DSS-induced mice IBD by reshaping the cell structure in colon mucosa

To explore the underlying mechanisms by which hADSC-Exo maintained the integrity of colon, we performed immunofluorescence staining experiments in colon tissues and examined several cell cerface markerss, such as Ki67 and β-catenin (specific markers of epithelial cell proliferation), EPH B2 and Lgr 5 (specific markers of intestinal stem cells) and Mucin (specific markers of goblet cells) hADSC-Exo treatment significantly increased the mean fluorescence intensity (MFI) of Ki67 (Fig. 3A, B) and β-catenin (Fig. 3C, D), EPH B2 (Fig. 3E, F) and Lgr 5 (Fig. 3G, H), and also Mucin (Fig. 3I, J), which indicated that hADSC-Exo significantly promoted the proliferation and regeneration of epithelial cell, intestinal stem cell and goblet cell in colon crypts. Collectively, hADSC-Exo could reshape the cell structure in colon mucosa to attenuate DSS-induced IBD.

### hADSC-Exo up-taken by Hcoepic cells and promotes wound healing of Hcoepic cells

In order to reveal the effect of hADSC-Exo on intestinal epithelial cells, we selected Hcoepic cell line as a research model and performed exosome up-taken experiments and wound healing assay DiI-labeled hADSC-Exo were added to the Hcoepic cell culture medium, and exosome uptake was assessed by fluorescence microscopy at different time points (4, 12, 24, and 48 h). The results indicated that hADSC-Exo can be up-taken by Hcoepic cell line from 4 h after DiI-labeled hADSC-Exo addition, and uptake reached a peak at 48 h (Fig. 4A, B).

Next, we performed wound healing assay to test the effect of hADSC-Exo on damage repair capacity and restitution of intestinal epithelial cell. hADSC-Exo (200 µg/ml) were added into FBS-free medium after scratching and photographed by microscopy at different time points (0, 4, 6, 12, 24, and 48 h). The results showed that the wound closure of Hcoepic cells at 48 h post-injury was strongly promoted by hADSC-Exo compared to that in control without exosomes (Fig. 4C, D).

### hADSC-Exo facilitates Hcoepic cells proliferation, promotes cell cycle and inhibits cell apoptosis

To further explore the function of hADSC-Exo on intestinal epithelial cells, we performed CCK8 assay, cell cycle assay and cell apoptosis experiment. First, we treated Hcoepic cells with different concentrations of hADSC-Exo for 24 h (Fig. 5A) and 48 h (Fig. 5B) and then detected the cell proliferation with CCK8 kit. hADSC-Exo could promote Hcoepic cells growth, even in a very low concentration. The results of cell cycle assay indicated that hADSC-Exo significantly increased the number of Hcoepic cells in S phase after 48h administration of hADSC-Exo (Fig. 5C). The results of cell apoptosis experiment indicated that hADSC-Exo dramatically reduced the proportion of apoptotic cells after 48 h administration compared with control group only with FBS free medium and without exosomes administration (Fig. 5D).

**Figure 7.**
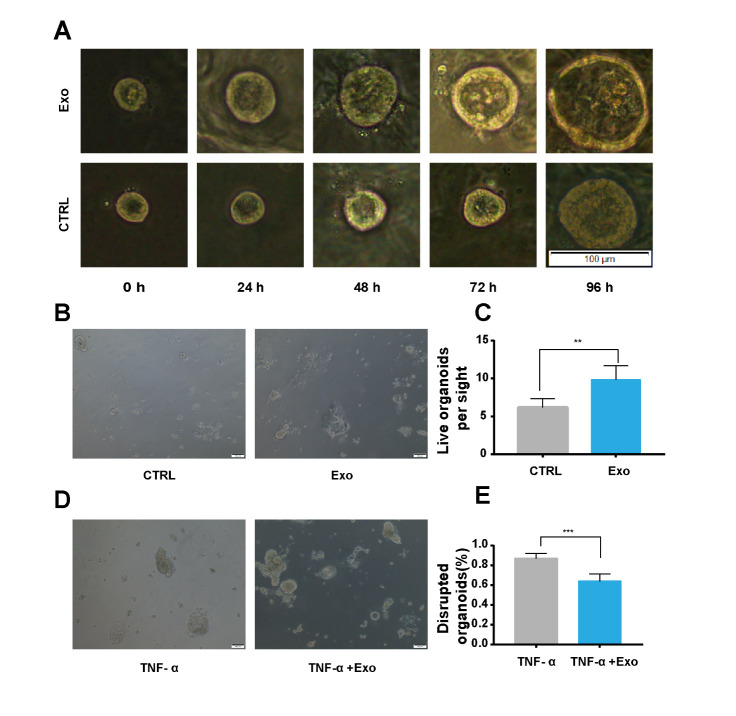
**hADSC-Exo increase the growth of colon organoids and endow colon organoids with properties to resist the inflammation damage induced by TNF-α**. (**A**) Size of organoids treated with/without hADSC-Exo (100 µg/ml), Exo was administrated from day 3 to day 7, 0 h, 24 h, 48 h, 72 h, 96 h. n=6 wells per group. Scale bar = 100 µm. (B and C) Numbers of live organoids treated with/without hADSC-Exo (100 µg/ml), Exo was administrated at day6 (72 h), n=6 wells per group. Scale bar = 100 µm. (D and E) Numbers of disrupted organoids, Exo administration 72 h later, colon organoids were treated with TNF-α (40 ng/ml), n=6 wells per group. Scale bar = 100 µm.

### hADSC-Exo increase the growth of colon organoids and endow colon organoids with properties to resist the inflammation damage induced by TNF-α

In order to further study the therapeutic effect of hADSC-Exo on mice IBD, we isolated crypts and single colon stem cells from mouse colon, and cultured colon organoid (Fig. 6C). We established a co-cultured system by co-culturing colon organoids, TNF-α (40 ng/ml) and hADSC-Exo together (Fig. 6A, B). On the third day after seeding organoids in matrigel, we added hADSC-Exo (100 µg/ml) into growth medium to explore the function of exosome on colon organoids regeneration and proliferation, and the results showed that hADSC-Exo increased the growth of colon organoids (Fig. 7A). Similarly, On the 6^th^ day after seeding organoids in matrigel, we added hADSC-Exo (100 µg/ml) into growth medium, 72 hours later, we observed the status of colon organoids by microscopy. The results showed that hADSC-Exo increased the growth of colon organoids in the exosome exposure group compared with the control group without hADSC-Exo (Fig. 7B, C). hADSC-Exo significantly reduced the numbers of disrupted colon organoids compared with that group only with TNF-α and without hADSC-Exo administration (Fig.7D, E).

## DISCUSSION

Inflammatory bowel disease (IBD) is a kind of gastrointestinal inflammatory disease with unknown etiology at present, and the incidence of IBD is increasingly rapidly all over the world [37, 38]. Patients with IBD have the risk of recurrence all their life. Severe patients are often not easy to recover and have poor prognosis. IBD has become a heavy burden for both patients and the society [37, 39]. Currently, the conventional therapeutic options for IBD including immunomodulators, thiopurine agents, anti-TNF-α monoclonal antibodies are still not satisfactory due to their undesirable side effects [40]. Therefore, designing more efficient therapeutic agents with few side effects has been the subject of intense investigation. In this study, we selected hADSC-Exo as a therapeutic agent for IBD and aimed to investigate the treatment efficacy of hADSC-Exo in IBD mouse and also to investigate the underlying mechanisms. Here, we found that hADSC-Exo was sufficient to alleviate the severity of IBD in mice by regulating inflammatory cytokines and reshaping cell constitution of colon in mice. At the same time, hADSC-Exo could increase the growth of colon organoids and endow colon organoids with properties to resist the inflammation damage induced by TNF-α.

Exosomes are extracellular vesicles (diameter: 20 - 200 nm) that share some of the characteristics and physiological state of their parent cells. They could deliver a wide array of biological molecules, such as nuclear acids, proteins and lipids to the target cells to perform their functions. Exosomes derived from different cells carry diverse bio-substances, and they perform different functions. Exosomes can be used for diagnosis, drug delivery, and as therapeutic agents [33, 41, 42]. One recent study reported that exosomes derived from intestinal epithelial cells containing annexin A1 contributed to wound repair of intestinal epithelium [43]. Another research provided evidence that fibroblast-derived exosomes delivered amphiregulin with Wnt and EGF activity to maintain intestine stem cell phenotype [30]. Tao et al. found that exosomal miR-208b from colorectal cells promoted Tregs expansion and related oxaliplatin resistance [44]. Li et al. reported that exosomal miRNA-19b-3p from tubular epithelial cells contributed to M1 macrophage activation in kidney damage [45]. A recent research showed that Pancreatic ductal adenocarcinoma derived exosome carried CD44v6/ C1QBP complex to promote pancreatic cancer liver metastasis [46]. Ju et al showed that grape exosome-like nanoparticles gradually accumulated in the gut 6 hours after administration [20]. Mao et al. [47] and Wang et al. [33] reported that exosomes derived from human umbilical cord MSCs could home to the IBD mice intestine and colon after 12 h of exosome injection. Consistent with their results, we also found that hADSC-Exo can be up-taken by IBD mice and can home to the inflammatory site of colon. Therefore, we speculate that exosomes perform their functions by homing to site of injury and delivering functional substances to the target cells.

It has been demonstrated that the levels of inflammatory cytokines (TNF-α, IL-1β, IL-6, IL-10 IL-13 and so on) play a crucial role in the pathogenesis of DSS-induced IBD and correlate with the degree of inflammation. Zhang et al. demonstrated that edible ginger-derived nanoparticles reduced the expression of pro-inflammatory cytokines (TNF-α, IL-6 and IL-1β), and increased the expression of anti-inflammatory cytokines (IL-10 and IL-22) in induced colitis [5]. Liu et al. found that MSC-Exo reduced murine colonic inflammation by increasing the levels of IL-10 and IL-7 [6]. Artemisinin analogue SM934 significantly inhibited both mRNA and protein expression of IL-6, IL-1βand TNF-α in the colon tissues of UC mice. Here, in our study, we illustrated that hADSC-Exo decreased the expression of IL-6, IL-1β, TNF-α, IL-12 and increased the expression of IL-13 in mice serum, and in the colon tissue, hADSC-Exo decreased the expression of IL-6 and increased the expression of IL-10.

The degree of intestinal damage is highly correlated with the integrity of intestinal mucosa whose maintenance requires the delicate balance between colonic stem cell renewal and intestinal epithelial cell proliferation [48]. Previous study demonstrated that the integrity of mucosa epithelial function played a key roles in etiology and pathogenesis of IBD, and that the intestinal stem cells (ISCs) governed the proliferation and regeneration of intestinal epithelium under certain conditions [43, 49]. Hou et al. revealed that IL-22 accelerated proliferation of intestinal epithelial, recovered damaged intestinal mucosa and ameliorated DSS- induced colitis in mice [36]. In our present study, we detected some specific markers in cell surface, such as Ki67, β-catenin, Mucin and Lgr5. We found that hADSC-Exo significantly promoted the proliferation and regeneration of intestinal stem cell and goblet cell and further influenced epithelial cell. Collectively, hADSC-Exo could reshape the cell structure in colon mucosa to attenuate DSS-induced IBD. We further selected intestinal epithelium cell line (Hcoepic) as an experimental model to evaluate the effects of hADSC-Exo in intestinal epithelium cells. Our results demonstrated that hADSC-Exo could be taken up by Hcoepic cells and promote the proliferation and migration of Hcoepic cells.

In recent years, organoids have shown great potential and application value in disease research. Small intestinal organoids and colon organoids are cultured with Lgr 5^+^ intestine stem cells isolated from intestine/colon crypts and can be selected as model for IBD [50, 51]. Ju et al. showed that grape-like nanoparticles were taken up by mouse intestinal stem cells and promoted the proliferation of Lgr5^+^ intestinal stem cells [52]. By use of humanized mouse intestinal organoid as in vitro IBD model, researchers found that LRH-1 maintained intestinal epithelial homeostasis and mitigated inflammatory injury [4]. In our present research, we found that hADSC-Exo also played pivotal role in promoting the proliferation of colonic organoids and endowed colonic organoids with the ability to resist inflammatory injury.

Taken together, our data showed that hADSC-Exo could ameliorate DSS-induced mouse IBD and improve the survival status of IBD mice significantly. hADSC-Exo can facilitate Hcoepic cells proliferation by promoting cell cycle and inhibiting cell apoptosis. hADSC-Exo reshaped the cell structure in colon crypts of DSS-induced mice IBD by the promotion of Lgr5+ ISCs, epithelial cells and goblet cells proliferation and regeneration. hADSC-Exo increased the growth of colon organoids and endowed colon organoids with properties to resist the inflammation damage induced by TNF-α. Therefore, hADSC-Exo significantly increased the number of living organoids and decreased the number of disrupted organoids induced by TNF-α. Overall, our results provide further evidence that hADSC-Exo may represent a novel treatment approach for inflammatory bowel disease.

## Supplementary Materials

The Supplementar data can be found online at: www.aginganddisease.org/EN/10.14336/AD.2021.0601.


