# Supplementary material for: Human Adipose Mesenchymal Stem Cell-derived Exosomes Protect Mice from DSS-Induced Inflammatory Bowel Disease by Promoting Intestinal-stem-cell and Epithelial Regeneration
Source: Aging Dis. 2021 Sep 1;12(6):1423–37. doi: 10.14336/AD.2021.0601 (PMC8407880; doi:10.14336/AD.2021.0601)
Supplement: Supplementary file 1 [file AD-12-6-1423-s.pdf]

SUPPLEMENTARY DATA

Human Adipose Mesenchymal Stem Cell-derived Exosomes Protect Mice from DSS-Induced Inflammatory Bowel Disease by Promoting Intestinal-stem-cell and Epithelial Regeneration

Hongliang Yu[1], Xudong Yang[1], Xian Xiao[1], Meiqian Xu[1], Yanlei Yang[1], Chunling Xue[1], Xuechun Li[1], Shihua Wang[1*], Robert Chunhua Zhao[1,2*]

© 2021. Yu H et al. Published online at http://www.aginganddisease.org/EN/10.14336/AD.2021.0601

SUPPLEMENTARY DATA

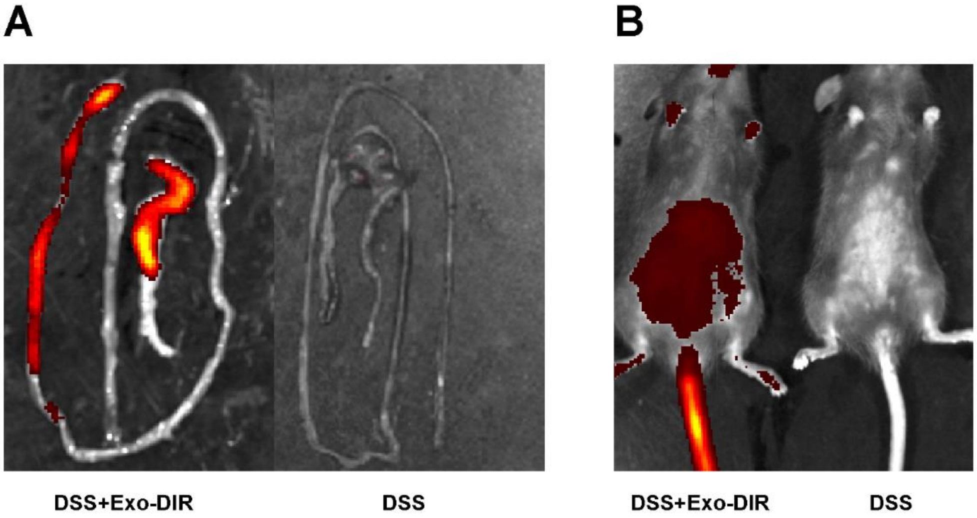

Supplementary Figure 1. Biodistribution of DIR-labeled hADSC-Exo in mice. (A) Fluorescence distribution of DIR-labeled hADSC-Exo in mice intestine tract and colon. **(B)** Fluorescence distribution of DIR-labeled hADSC-Exo in mice abdominal cavity.

© 2021. Yu H et al. Published online at http://www.aginganddisease.org/EN/10.14336/AD.2021.0601
